# A 12-week double-blind randomised controlled trial investigating the effect of dietary supplementation with 125 *μ*g/d vitamin D in adults with asthma

**DOI:** 10.1017/S0007114524000953

**Published:** 2024-09-28

**Authors:** Stephanie Watkins, Tanja Harrison, Sohail Mushtaq

**Affiliations:** Faculty of Health, Medicine and Society, University of Chester, Chester CH1 4BJ, UK

**Keywords:** Vitamin D, Asthma, Lung function, Dietary supplementation

## Abstract

Vitamin D deficiency has previously been linked to higher rates of exacerbation and reduced lung function in asthmatics. Previous randomised controlled trials investigating the effect of vitamin D supplementation have mainly focused on children with asthma. Trials involving adults have typically used bolus dosing regimens, and the main outcomes have been patient-focused without investigating underlying inflammation. The present study aimed to conduct a 12-week placebo-controlled randomised controlled trials administering a daily 125 µg vitamin D_3_ supplement to adults with mild to moderate asthma. A total of 32 participants were randomised to receive either the 125 μg vitamin D_3_ supplement or an identical matching placebo. The primary outcome of the study was lung function measured by the ratio of FEV_1_:FVC (effect size 2·5) with secondary outcomes including asthma symptoms and inflammatory biomarkers. There was a small but statistically significant higher increase in the mean (±sd) ratio of FEV_1_:FVC from baseline to post-intervention in the vitamin D group (+0·05 ± 0·06) compared with the placebo group (+0·006 ± 0·04, *P* = 0·04). There was no effect of the intervention on asthma control test scores, or the inflammatory biomarkers measured. There was a moderate, significant association between baseline plasma 25(OH)D concentration and baseline plasma IL-10 (*r* = 0·527, *P* = 0·005) and TNF-*α* (*r* = −0·498. *P* = 0·008) concentrations. A daily vitamin D_3_ supplement led to slightly improved lung function in adult asthmatics and may be a useful adjunct to existing asthma control strategies, particularly for individuals with suboptimal vitamin D status.

## Graphical abstract

**Figure:**
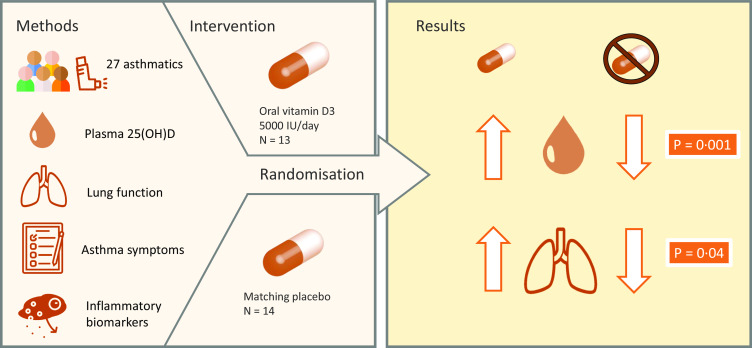

Vitamin D is a secosteroid hormone^(1)^ with the classical role of increasing the efficiency of Ca and phosphorous absorption^(2)^. Vitamin D has also been shown to play an important role in diseases, including cancer^(3)^, diabetes^(4)^ and respiratory diseases such as asthma^(5)^.

Asthma is a disease defined by the chronic inflammation of the airways, which causes bronchial hyper-reactivity^(6)^, overproduction of mucus^(7)^, remodelling of the airway wall and narrowing of the airways^(8)^. In patients with the disease, this leads to symptoms including repeated periods of shortness of breath, wheezing and the feeling of a tight chest. Current data show that one in twelve adults and one in eleven children are currently receiving treatment for asthma in the UK, with the UK having some of the highest prevalence in Europe^(9)^.

Vitamin D deficiency is becoming more common in developed countries^(10,11)^. In the UK, 30–40 % of the general population are classed as vitamin D-deficient (serum or plasma 25(OH)D concentration < 25 nmol/l) during the winter^(12)^. A reference nutrient intake for vitamin D was proposed by the Scientific Advisory Committee on Nutrition (SACN) of 10 μg/d^(13)^. Vitamin D deficiency has previously been linked to asthma with more consistent findings of a link in children^(14–17)^ than in adults^(18–20)^. In children and adults, vitamin D insufficiency has been associated with reduced lung function^(18)^. Thresholds for vitamin D deficiency, however, are inconsistent across studies with some defining deficiency as a serum or plasma 25(OH)D concentration as < 25 nmol/l^(19)^, < 30 nmol/l^(14)^, < 50 nmol/l^(15–17)^ and less than < 75 nmol/l^(18,20)^. Systematic reviews and meta-analyses to evaluate the efficacy of vitamin D supplementation and effect of vitamin D concentrations on outcomes in patients with asthma have largely focused on children^(21–24)^. There have been few systematic reviews and meta-analyses investigating the potential effect in adults, although these have been promising, showing that vitamin D supplementation significantly reduces the rate of asthma exacerbations in patients with asthma^(5,25)^.

The immunomodulatory effects of vitamin D are significant in many diseases^(26)^. Due to its role in these diseases, vitamin D supplementation is being explored as a potential therapy. Asthma is described as the hallmark Th2 disorder of the lungs supported by findings that asthmatic airway inflammation is eosinophilic in nature^(27)^ and in the asthma disease state there are an increased number of CD4^+^ T cells producing IL-4 and IL-5^(28)^. The balance between Th2 and Th1 immunity is important in asthma. Induction of the Th1 response by IL-12 or transfer of Th1 cells suppresses the expression of Th2 cytokines and associated asthma responses, including airway hyperresponsiveness and inflammation in mouse models^(29)^. Furthermore, mice lacking the Th2 cytokine IL-4 had reductions in eosinophilia and airway inflammation^(30)^. IL-13 is key in bronchial hyper-reactivity and goblet cell metaplasia and contributes to the pathophysiological features of asthma independently of Ig-E and eosinophils^(31)^ representing a potential therapeutic treatment for asthma. Vitamin D has a better binding capacity to IL-13 than the common asthma medication mometasone^(32)^, which suggests that vitamin D could be used for increased inhibition of IL-13 in asthmatics leading to a reduced inflammatory response. It has been proposed that vitamin D could afford protection through its anti-inflammatory capabilities. Initial *in vitro* research investigating the effect of vitamin D on immune cells found that the addition of calcitriol to T cells inhibited the production of the inflammatory cytokines interferon-γ (IFN-*γ*), IL-17 and IL-21^(33)^. Respiratory epithelial cells can convert 25(OH)D into active calcitriol, which reduces expression of the pro-inflammatory cytokine IL-8^(34)^.

The aim of the present study was to investigate the effect of a daily 125 µg vitamin D supplement on asthma in adults aged 18–65 years over 12 weeks on lung function, asthma control and inflammatory biomarkers and, furthermore, to examine baseline plasma 25(OH)D concentrations in adults with asthma and the efficacy of the supplement in improving vitamin D status.

## Methods

### Study design

Adults with mild to moderate asthma were recruited from the University of Chester and surrounding areas. Eligibility for the study was assessed by participants filling out an online screening questionnaire. Eligible participants were adults between the age of 18 and 65 years, who have had asthma diagnosed by a general practitioner for at least 12 months and require inhaled asthma therapy at least twice a week. This is in line with the National Asthma Education and Prevention Program definition of mild to moderate asthma^(35)^. Participants were excluded if they were unable to communicate in English, already taking a supplement containing vitamin D, had suffered a respiratory tract infection within the past 4 weeks, had a history of asthma requiring intubation or mechanical ventilation, pregnant women, smokers and those who had recently been on a sun holiday or planned to go on a sun holiday during the trial period.

A total of seventy-four subjects completed the screening questionnaire, and fifty were eligible based on the inclusion criteria. However, fifteen of the eligible subjects did not respond to invitations to attend a clinic, and three of the eligible subjects did not attend their first clinic. After screening, thirty-two subjects were included and randomised to the intervention. During the study, five participants withdrew. The reasons given for dropout were not able to attend follow-up session due to COVID-19 pandemic (*n* 2), not taking supplements (*n* 2) and no reason given (*n* 1). A total of twenty-seven participants completed the study and were included in the analysis.

**Fig. 1.**
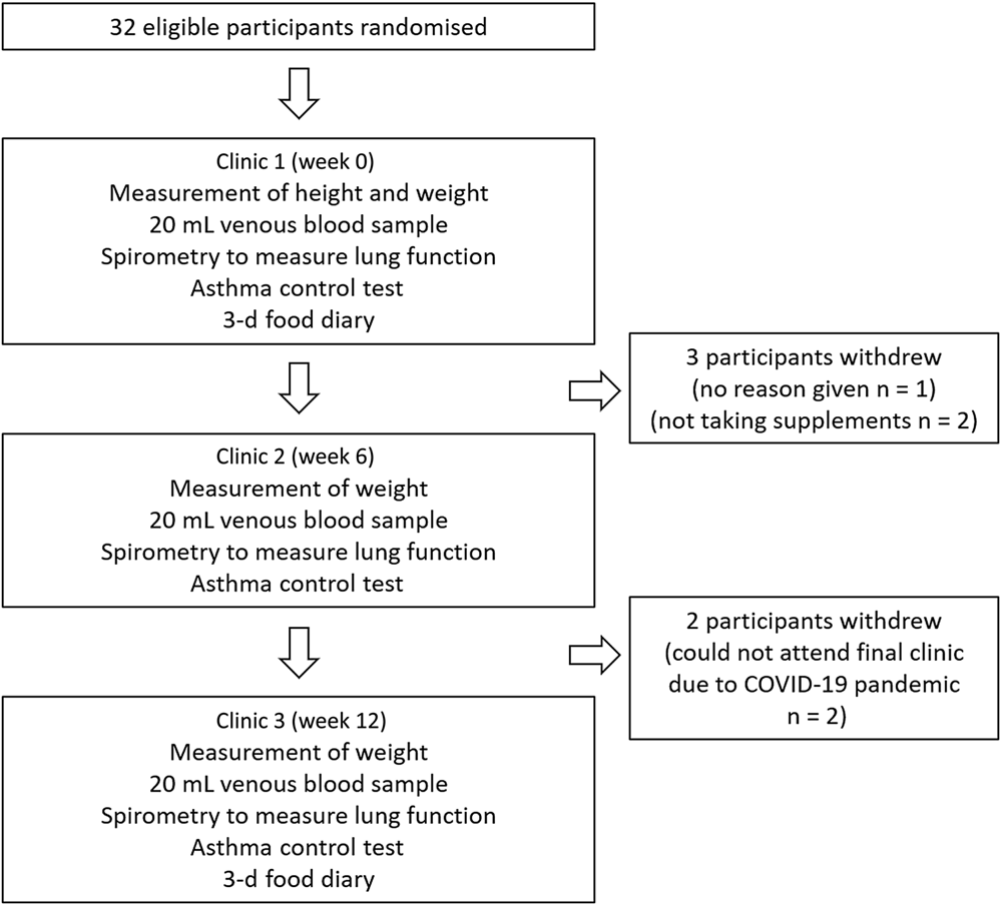
Flow chart of study protocol.

The two data collection phases were between November 2019–March 2020 and November 2021–March 2022 as these months are periods where vitamin D cannot be synthesised endogenously in the UK. Data collection was not possible during the winter of 2020−2021 due to the COVID-19 pandemic. Following screening, participants entered the intervention phase. The randomisation protocol was carried out using computer software (randomisation.com) by an independent, third party. Subjects (*n* 32) were block-randomised in pairs to two groups: vitamin D group (125 μg, BioTech Pharmacal Inc.) or placebo group (identical matching capsule custom-made by BioTech Pharmacal). The dosing regimen was chosen as a dose that would reasonably increase plasma 25(OH)D concentrations over a 3-month winter period in a population that have a high prevalence of vitamin D insufficiency and deficiency^(20)^. The third party allocated ninety capsules of vitamin D or placebo into identical tamper-proof supplement bottles. An excess of six capsules in each bottle allowed the researcher to estimate compliance on completion of the study. Compliance to the supplementation (%) was estimated as follows: (total number of capsules [90] – remaining number of capsules)/84 × 100. The subjects and researcher were blinded to group assignment of each subject. The blinding was maintained throughout the study period of 12 weeks and was not unlocked until data analysis of all subjects was complete. Participants attended three clinics in total: baseline clinic, interim clinic at 6 weeks and final clinic at 12 weeks. The schematic study protocol can be seen in Fig. 1.

### Study protocols

Height (cm) was measured to the nearest 0·1 cm with a wall-mounted digital stadiometer (Model 264 SECA) at clinic one. Body weight (kg) was measured to the nearest 0·1 kg using electronic weighing scales (Model 875 SECA) at clinic one, two and three. BMI was calculated using height and weight obtained. The equation used to calculate BMI was BMI (kg/m^2^) = weight (kg)/height (m^2^).

Lung function was measured using a handheld spirometer (Vitalograph micro 6300) which gave measurements for forced expiratory volume in one second (FEV_1_), forced vital capacity (FVC), predicted FEV_1_ and predicted FVC. Subjects wore a nose clip to ensure the most accurate reading. Three measurements were taken with the highest value for FEV_1_, % predicted FEV_1_, FVC, % predicted FVC and FEV_1_:FVC used for the final outcomes.

The asthma control test^(36)^ was used to assess each subject’s perceived level of control of asthma symptoms. The questionnaire included questions on the amount of time asthma prevented productivity at work, school or in the home, how often subjects experienced shortness of breath, how often symptoms woke subjects during the night, how often reliever medication was used and a rating of asthma control. Scores were categorised with the maximum score of 25 indicating asthma appeared to have been under control, a score of 20–24 indicating asthma appeared to be reasonably well controlled and a score of less than 20 indicating asthma may not have been controlled.

Subjects were required to complete a 3-d food diary during the first and last weeks of the intervention (to include two weekdays and one weekend day) to estimate their habitual dietary intake of vitamin D. Dietary records were analysed for habitual vitamin D intake (µg/d) using Nutritics Professional Nutrition Analysis Software (Nutritics Ltd).

A venous blood sample was collected at clinic one, two and three. Blood was collected into vacutainers pre-sprayed with the anti-coagulant dipotassium EDTA (K2 EDTA). Both whole blood and plasma obtained from the venepuncture were used for the analysis. Whole blood was used to measure the full blood count using an automated haematology analyser (Beckman Coulter DxH 520). Venous blood samples were centrifuged for 15 min (1600 g) at 4°C to obtain plasma samples required to analyse vitamin D metabolism biomarkers (25(OH)D and parathyroid hormone (PTH)) and inflammatory biomarkers (C-reactive protein (CRP), TNF-*α*, IFN-*γ*, IL-4, IL-10, IL-13 and IgE). Plasma samples were aliquoted into microcentrifuge tubes and stored at −80°C until analysis was carried out.

Plasma samples were used for the analysis of biomarkers using commercially available kits: 25(OH)D (Aviva Systems Biology; OKBA00028), PTH (Abcam; ab230931), CRP (Abcam; ab99995), TNF-*α* (Invitrogen; KHC3011), IFN-*γ* (Abcam; ab100537), IL-4 (Abcam; ab215089), IL-10 (Invitrogen; 88–7106), IL-13 (Invitrogen; 88–7439) and IgE (Invitrogen; 88–50 610). Quality controls (low, medium and high) were used to determine intra- and interassay CV. The intra- and inter-assay CV for all biomarkers were as follows: 25(OH)D: 8·98 %, 10·21 %; PTH: 8·50 %, 12·22 %; CRP: 6·94 %, 8·11 %; IgE: 4·54 %, 4·66 %; IFN-*γ*: 11·23 %, 14·32 %; TNF-*α*: 7·70 %, 13·40 %; IL-4:12·35 %, 15·00 %; IL-10:3·04 %, 10·10 %; and IL-13:4·25 %, 4·32 %. For all biomarkers, standards and a blank were run in triplicate, and samples were run in duplicate. Optical density was measured on a microplate reader (Thermo Scientific MultiSkan FC) at 450 nm. All data were analysed using Thermo Scientific MultiSkan software. Resting values were multiplied by a sample dilution factor, if used, to provide the concentration of target biomarker in each sample. For samples that tested lower than the limit of detection without dilution, the value used for analysis was the lowest standard divided by 2^(37)^.

### Power calculation

Sample size was estimated using mean and standard deviation data from a study in which FEV_1_ post-intervention at 24 weeks of 82·5 ± 3·5 L (vitamin D treatment group) and 76 ± 2 L (placebo group) was reported in asthma patients receiving 100, 000 μg (2500 µg) bolus vitamin D plus 50, 000 μg (1250 µg) orally weekly^(38)^. With an effect size of 2·5 and power of 0·8, the total sample size required for each group in the present study was nine. Allowing for a 20 % dropout rate, and this study lasting only 12 weeks, the total sample size needed for the whole study was 28 (14 subjects/group). The sample size was estimated using G Power Software (version 3.1.9.4) and is based on data for the primary outcomes of the present study: lung function measured using FEV_1_.

### Statistical analysis

All statistical analyses were performed using IBM SPSS software (version 28). Data were checked for homogeneity of variance and normal distribution using the Shapiro–Wilks test. Baseline comparisons between the vitamin D and placebo groups were carried out using independent *t* test and Mann–Whitney test. Mixed model repeated-measures ANOVA was performed to determine the interaction with time points for all vitamin D biomarkers, inflammatory biomarkers and lung function data. *Post hoc* analyses were carried out when intervention × time point interactions were observed. Changes in all vitamin D biomarkers, inflammatory biomarkers and lung function measurements from baseline (week 0) to post-intervention (week 12), baseline (week 0) to interim (6 weeks) and interim (week 6) to post-intervention (week 12) between the two groups were compared and analysed. Pearson’s or Spearman’s correlation coefficient tests were used to investigate the relationship between plasma 25(OH)D concentrations and lung function or inflammatory measures. Statistical significance was accepted when *P* ≤ 0·05.

## Results

A total of thirty-two asthmatic adults commenced the study and were randomised to either the vitamin D or placebo group. At 12 weeks, twenty-seven participants completed the trial. Overall mean (±sd) compliance to supplementation was 93·7 ± 8·2 %. For the vitamin D group, mean (±sd) compliance was 94·2 ± 7·6 %. For the placebo group, mean (±sd) compliance was 92·4 ± 8·9 %. There were no adverse events reported during the trial period.

The baseline characteristics are reported based on the data available for the twenty-seven participants who completed the study. The participants recruited were aged between 20 and 62 years with a mean (±sd) age of 38·3 ± 12·9 years. Most participants were White (96·3 %) followed by South-East Asian (3·7%). The baseline characteristics of participants are summarised in Table 1. There were no significant differences in the height, weight, BMI or plasma 25(OH)D concentration between the vitamin D group and placebo group. There was a significant difference between the mean (±sd) dietary vitamin D intake in the vitamin D group (4·4 ± 3·9 µg/d) and the placebo group (1·7 ± 1·2 µg/d, *P* = 0·013).

**Table 1.** Baseline characteristics of participants

Characteristic	All (*n* 27)		Vitamin D group (*n* 13)		Placebo group (*n* 14)		*P*
	Mean	sd	Mean	sd	Mean	sd	
Sex (male = M, female = F)	M = 9, F = 18		M = 4, F = 9		M = 5, F = 9		n/a
Age (years)	38·3	12·9	35·0	13·0	41·0	12·0	0·275
Height (cm)	169·0	7·5	168·9	7·6	169·1	7·7	0·94
Weight (kg)	80·5	22·6	78·5	21·6	82·3	24·0	0·752
BMI (kg/m^2^)	28·0	7·0	27·2	6·0	28·8	8·0	0·716
Dietary vitamin D intake (µg/d)	3·0	3·1	4·4	3·9	1·7	1·2	0·013
Plasma 25(OH)D concentration (nmol/l)	36·8	15·2	36·7	13·2	36·0	17·9	0·872

All values are presented as mean and standard deviation.

*P*-value of ≥ 0·05 indicates no significant difference at baseline between the two groups.

The thresholds described by SACN^(13)^ were used to categorise participants as vitamin D sufficient (> 50 nmol/l), insufficient (25–50 nmol/l) or deficient (< 25 nmol/l) based on plasma 25(OH)D concentration at baseline. The prevalence of vitamin D sufficiency at baseline was 19 % (*n* 5), insufficiency was 59 % (*n* 16) and deficiency was 22 % (*n* 6). In the vitamin D group, one participant was unable to provide a blood sample for the measurement of vitamin D status and inflammatory biomarkers at week 6. In the placebo group, one participant was unable to provide a blood sample for the measurement of vitamin D status and inflammatory biomarkers at week 6 and week 12.

**Fig. 2.**
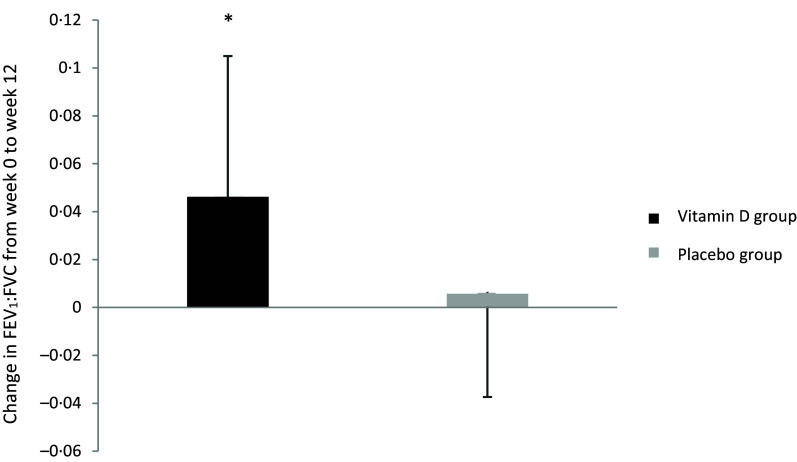
The effect of intervention on mean (±sd) change in FEV_1_:FVC from baseline (week 0) to post-intervention (12 weeks) in the vitamin D group and the placebo group, *P < 0·05. FEV1, forced expiratory volume in one second; FVC, forced vital capacity.

Table 2 shows the effect of the intervention on lung function and asthma control. The increase in the ratio of FEV_1_:FVC (+0·046 ± 0·059) in the vitamin D group from baseline (week 0) to post-intervention (week 12) was significantly higher than the increase in the placebo group (+0·006 ± 0·043, *P* = 0·040; Fig. 2). There was no effect of the intervention on the ratio of FEV_1_:FVC at any other time point. In the vitamin D group, daily supplementation with vitamin D did not impact other lung function parameters including FEV_1_ and FVC compared with the placebo group. There was also no effect of the intervention on asthma control test scores.

**Table 2.** Effect of intervention on clinical measures from baseline (week 0) to post-intervention (week 12) (*n* 27)

Measure	Vitamin D group (*n* 13)						Placebo group (*n* 14)						*P*
	Baseline		Post-intervention		Change		Baseline		Post-intervention		Change		
	Mean	sd	Mean	sd	Mean	sd	Mean	sd	Mean	sd	Mean	sd	
FEV_1_ (L)	2·93	0·83	2·86	0·87	–0·07	0·33	2·92	0·63	2·97	0·74	+0·04	0·56	0·734
FEV_1_ (% predicted)	86·85	16·71	84·54	16·45	–2·31	9·56	91·64	22·79	91·07	21·69	–0·57	18·03	0·760
FVC (L)	3·39	0·81	3·16	0·90	–0·24	0·34	3·17	0·79	3·27	0·88	+0·10	0·68	0·152
FVC (% predicted)	87·46	17·09	80·85	19·35	–6·62	8·71	86·00	22·96	84·21	22·14	–1·79	17·13	0·371
FEV_1_:FVC	0·86	0·14	0·91	0·11	+0·05	0·06	0·92	0·11	0·92	0·12	+0·006	0·04	0·040
Asthma control test score	19·62	4·93	21·38	3·25	+1·77	4·23	18·43	2·50	18·86	4·42	–0·43	5·26	0·679

FEV_1_, forced expiratory volume in one second; FVC, forced vital capacity.

All values are presented as mean and standard deviation.

Changes are from baseline to post-intervention (week 0–week 12).

*P*-value represents the difference in changes between group 1 and group 2.

Intention-to-treat analysis using a ‘last observation’ single imputation was carried out on the change in outcomes FEV_1_, FVC and ratio FEV_1_:FVC from baseline to post-intervention between the vitamin D intervention and placebo group. There was no significant difference in change in FEV_1_ (*P* = 0·763), FVC (*P* = 0·300) or FEV_1_:FVC (*P* = 0·093) from baseline to post-intervention between the vitamin D and placebo group.

Table 3 shows the effect of the intervention on participants’ vitamin D status biomarkers at baseline, interim and post-intervention time points. As expected, plasma 25(OH)D concentration was significantly higher in the vitamin D group compared with the placebo group at 6 weeks and post-intervention. In the vitamin D group, the prevalence of vitamin D sufficiency at post-intervention was 38·5 % (*n* 5), insufficiency was 61·5 % (*n* 8) and deficiency was 0 % (*n* 0). In the placebo group, the prevalence of vitamin D sufficiency at post-intervention was 0 % (*n* 0), insufficiency was 38·5 % (*n* 5) and deficiency was 61·5 % (*n* 8).

**Table 3.** Effect of vitamin D supplementation on vitamin D status biomarkers at three time points over 12-week intervention period

Biomarker	Group	Baseline (week 0)		Interim (week 6)		Post-intervention (week 12)		Time (*P*)	Time × group (*P*)
		Mean	sd	Mean	sd	Mean	sd		
Plasma 25(OH)D (nmol/l)	Vitamin D	36·72	13·21	44·11	14·03	47·88	9·62	<0·0001	0·257
	Placebo	36·04	17·87	21·73	12·81	19·78	11·86		
Plasma PTH (pg/ml)	Vitamin D	8·29	8·27	7·41	7·61	4·82	3·78	0·026	0·395
	Placebo	12·37	11·36	9·31	5·79	9·68	7·09		

PTH, parathyroid hormone.

All values are presented as mean and standard deviation.

Significance accepted at *P* ≤ 0·05.

**Fig. 3.**
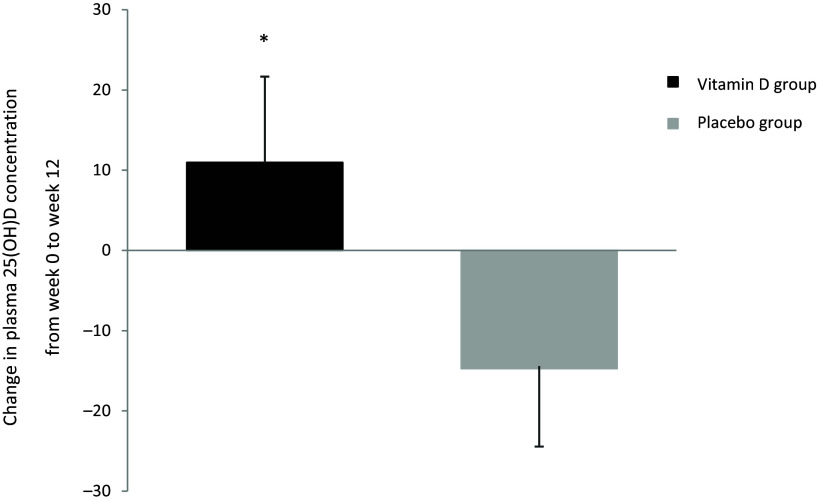
The effect of intervention on mean (±sd) plasma 25(OH)D concentration from baseline (week 0) to post-intervention (12 weeks) in the vitamin D group and the placebo group, *P < 0·01.

Figure 3 shows that the increase from baseline to post-intervention in plasma 25(OH)D concentration in the vitamin D group (+10·97 nmol/l) was significantly higher than the change in the placebo group (–14·73 nmol/l, *P* = 0·001). The increase from baseline to 6 weeks in plasma 25(OH)D concentration in the vitamin D group (+7·39 nmol/l) was also significantly higher than the change in the placebo group (–14·31 nmol/l, *P* = 0·001). The increase at post-intervention from 6 weeks in the vitamin D group (+3·77 nmol/l) was not significantly higher than the change in the placebo group (–0·42 nmol/l, *P* = 0·115). Mean (±sd) plasma 25(OH)D concentration was categorised as insufficient (25–50 nmol/l) at baseline and whilst higher at 6 weeks and post-intervention, it was still considered insufficient in the vitamin D group. However, the mean (±sd) plasma 25(OH)D concentration was considered insufficient at baseline but was deficient (< 25 nmol/l) in the placebo group by week 6 and week 12. In the vitamin D group, mean (±sd) plasma PTH concentration was significantly lower at post-intervention (4·82 ± 3·78 pg/ml, *P* = 0·039) compared with baseline (8·29 ± 8·27 pg/ml) but not significantly lower at 6 weeks (7·41 ± 7·61 pg/ml, *P* = 0·182). No significant difference in plasma PTH concentration was observed within the placebo group between each time point.

In the vitamin D group, daily supplementation with vitamin D did not have an impact on the change in granulocyte number compared with the placebo group. There was also no effect of the intervention on the change in any of the inflammatory biomarkers measured between the vitamin D group and placebo group (Table 4). All values for IL-13 were below the limit of detection.

**Table 4.** Effect of intervention on granulocyte numbers and plasma biomarkers from baseline to post-intervention (*n* 26)

Biomarkers	Vitamin D group						Placebo group						*P*
	Baseline (*n* 13)		Post-intervention (*n* 13)		Change		Baseline (*n* 14)		Post-intervention (*n* 13)		Change		
	Mean	sd	Mean	sd	Mean	sd	Mean	sd	Mean	sd	Mean	sd	
Whole blood granulocyte number (×10^9^/l)	4·17	1·59	3·54	1·39	–0·62	0·90	4·06	1·45	4·24	2·57	–0·18	1·99	0·329
Plasma IL-4 (ng/l)	172·00	136·17	173·99	219·09	2·00	159·43	185·49	133·67	132·28	204·14	–53·21	252·13	0·150
Plasma IFN-*γ* (pg/ml)	3·51	2·77	5·22	4·04	1·70	5·23	4·24	3·42	5·53	3·78	1·29	4·50	0·837
Plasma TNF-*α* (pg/ml)	42·52	66·18	21·03	29·16	–21·09	68·29	33·98	59·16	18·36	15·22	–15·63	60·87	0·343
Plasma IgE (μg/ml)	695·10	498·43	625·29	548·63	–69·81	236·57	652·25	562·51	389·09	381·49	–263·15	401·54	0·427
Plasma IL-10 (pg/ml)	9·46	13·82	3·14	3·29	–6·47	10·78	2·99	3·20	5·55	8·66	2·32	3·92	0·369
Plasma CRP (mg/l)	5·73	4·98	7·33	5·56	1·60	8·12	4·95	3·00	6·59	4·73	1·64	6·32	0·989

IFN, interferon; CRP, C-reactive protein.

All values are presented as mean and standard deviation.

Changes are from baseline to post-intervention (week 0–week 12).

*P*-value represents the difference in changes between group 1 and group 2.

**Fig. 4.**
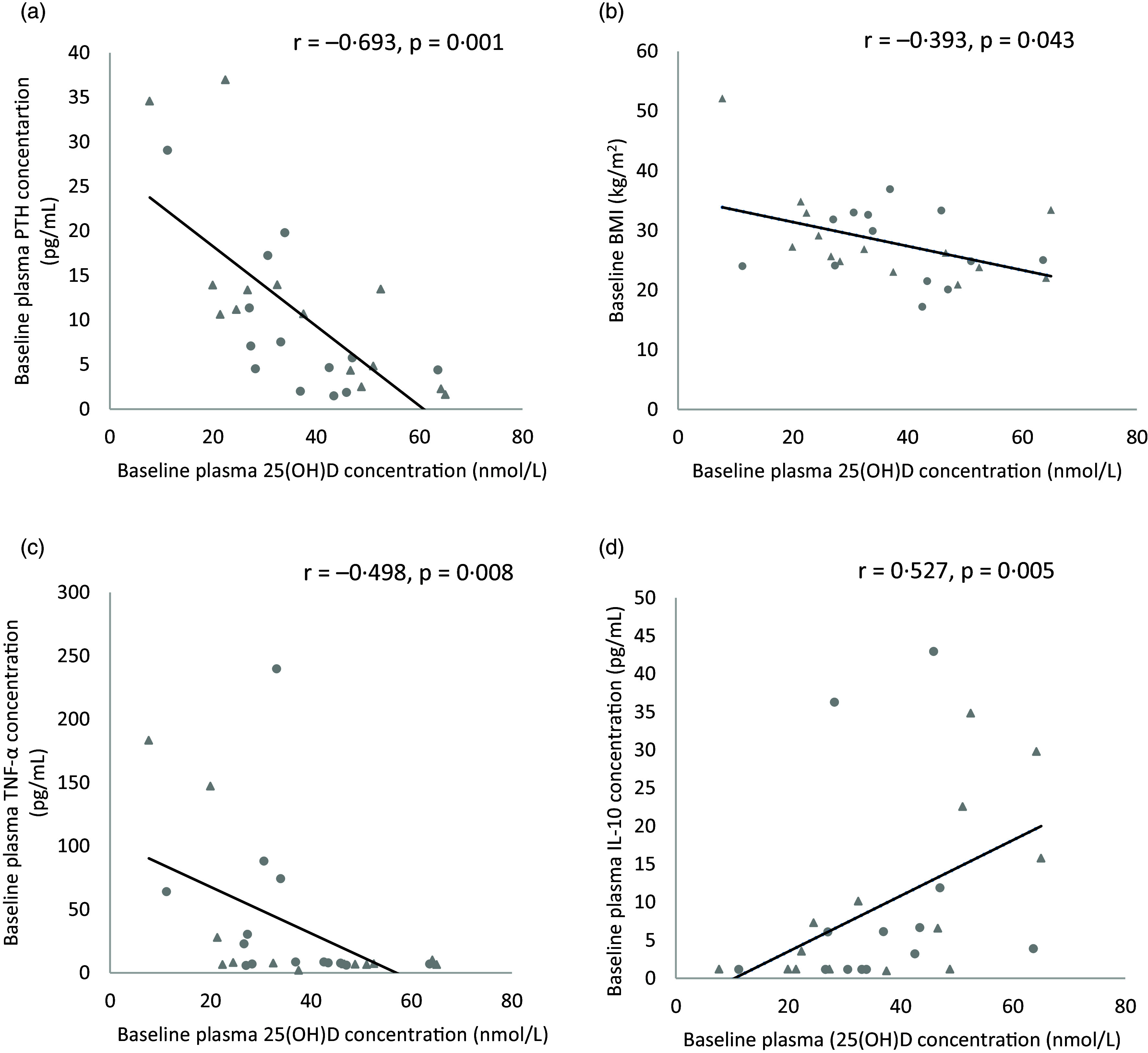
Association between baseline plasma 25(OH)D concentrations and baseline: (a) PTH concentration, (b) BMI, (c) TNF-*α* concentration and (d) IL-10 concentration in the combined vitamin D and placebo groups (*n* 27). Data points for the vitamin D group are denoted by a circular marker, and data points for the placebo group are denoted by a triangle marker. PTH, parathyroid hormone.

As shown in Fig. 4, a strong significant inverse association was observed between baseline plasma 25(OH)D concentrations and baseline plasma PTH concentration (*r* = −0·693, *P* = 0·001). A weak but significant inverse association was also found between baseline plasma 25(OH)D concentration and baseline BMI (*r* = −0·393, *P* = 0·043). Furthermore, a moderate, significant association was observed between baseline plasma 25(OH)D concentration and baseline plasma TNF-*α* levels (*r* = −0·498, *P* = 0·008). A moderate, significant positive association was found between baseline plasma 25(OH)D concentrations and baseline plasma IL-10 levels (*r* = 0·527, *P* = 0·005).

**Fig. 5.**
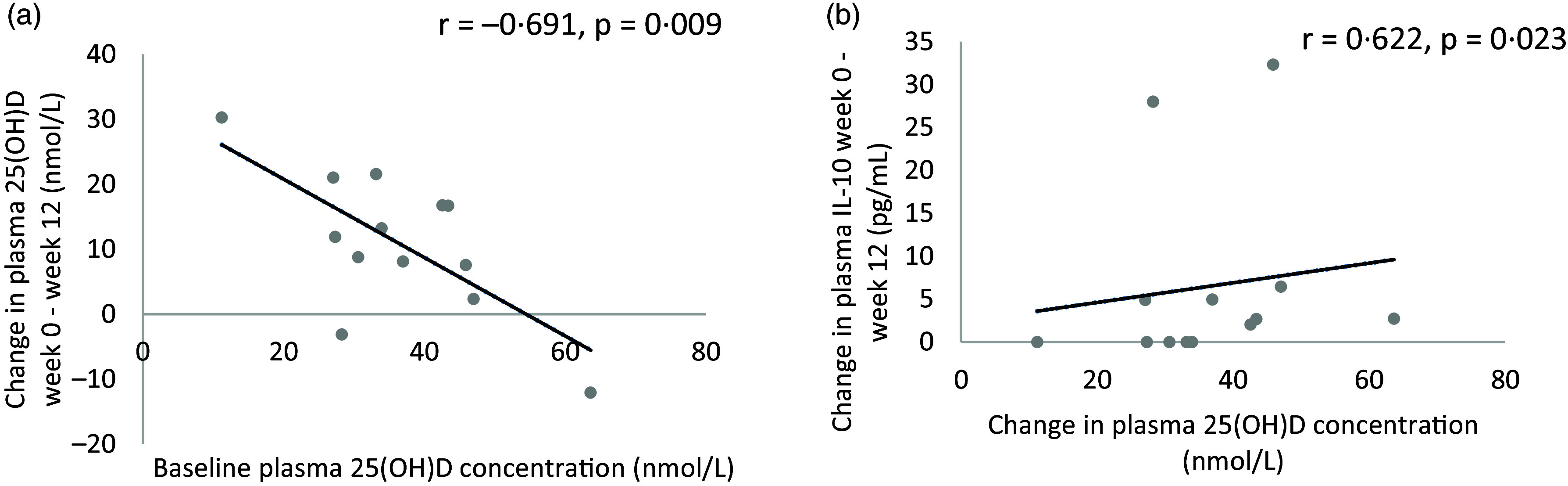
Association between (a) baseline plasma 25(OH)D concentration and the changes (week 0–week 12) in plasma 25(OH)D concentration, (b) change in plasma 25(OH)D concentration from week 0 to week 12 and change in plasma IL-10 concentration in the vitamin D group (*n* 13).

In the vitamin D group (*n* 13), there was a strong, significant inverse association between baseline plasma 25(OH)D concentration and the mean changes (week 0–week 12) in plasma 25(OH)D concentration (*r* = −0·691, *P* = 0·009; Fig. 5). To determine the potential effect of the improvement in vitamin D status in the vitamin D group on participant’s outcomes, correlations were carried out between the mean changes (week 0–week 12) in plasma 25(OH)D concentration with mean changes of all outcomes. There was a strong, significant association between mean change in plasma 25(OH)D concentration from week 0 to week 12 and mean change in plasma IL-10 concentration from week 0 to week 12 (*r* = 0·622, *P* = 0·023; Fig. 5).

**Fig. 6.**
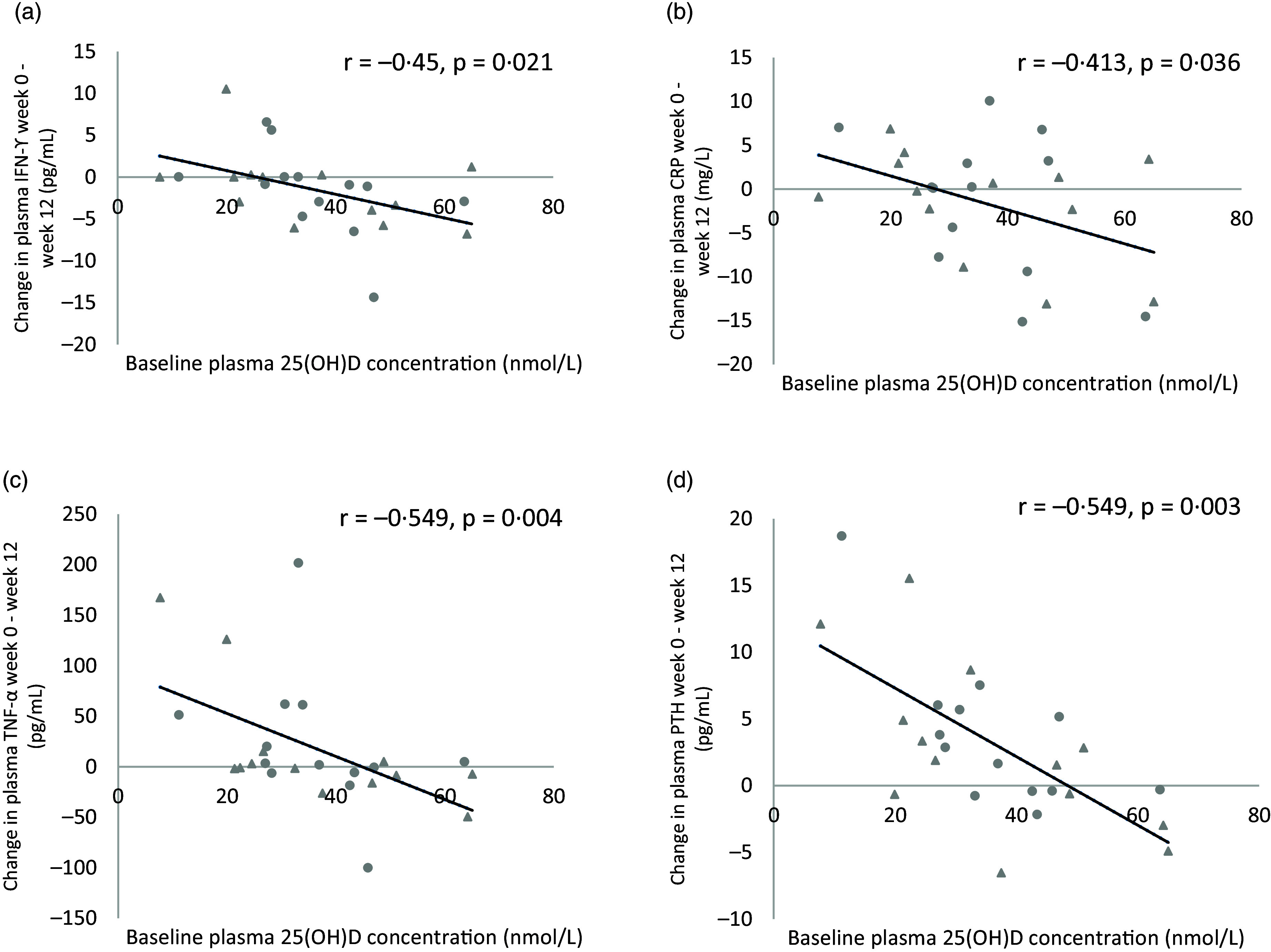
Association between baseline plasma 25(OH)D concentrations and mean difference in (a) IFN-*γ* concentration, (b) CRP concentration, (c) TNF-*α* concentration and (d) PTH concentration from week 0 to week 12 in both groups combined (*n* 26). Data points for the vitamin D group are denoted by a circular marker, and data points for the placebo group are denoted by a triangle marker. IFN-*γ*, interferon-*γ*; CRP, C-reactive protein; PTH, parathyroid hormone.

For the total sample, there was a moderate, significant inverse association between baseline plasma 25(OH)D concentration and the difference (week 0–week 12) in plasma IFN-*γ* concentration (*r* = −0·45, *P* = 0·021), plasma CRP concentration (*r* = −0·413, *P* = 0·036), plasma TNF-*α* concentration (*r* = −0·549, *P* = 0·004) and plasma PTH concentration (*r* = −0·549, *P* = 0·003; Fig. 6). No significant association was observed between baseline plasma 25(OH)D concentration and the difference in asthma control test score, ratio of FEV_1_:FVC, granulocyte number, plasma IL-4 concentration, plasma IgE concentration or plasma IL-10 concentration from week 0 to week 12.

## Discussion

The present study investigated the effect of 12-week dietary supplementation with a 125 µg daily dose of vitamin D on lung function and symptoms in adults with asthma. The study aimed to determine the effect of vitamin D as a potential addition to current asthma treatments as well as the efficacy of the dosing strategy at elevating plasma 25(OH)D concentrations in asthmatics.

As expected, plasma 25(OH)D concentration increased from baseline to post-intervention in the vitamin D group and was significantly higher than in the placebo group. In the placebo group, plasma 25(OH)D concentration decreased significantly from baseline to post-intervention. This shows that the vitamin D supplementation used in the study was effective in improving participants’ vitamin D status from baseline to post-intervention. While this increase was statistically significant, it was lower than expected, as the mean 25(OH)D concentration remained in the insufficient levels category based on the thresholds proposed by SACN (2016). In a previous intervention study, it was found that, for asthmatics, baseline serum 25(OH)D levels were associated with vitamin D sufficiency following 12 weeks of supplementation with 2500 μg of vitamin D once and then 100 μg/d for 28 weeks. Therefore, it may be necessary for supplementation regimens to use a bolus dose to initially increase plasma 25(OH)D levels before commencing daily dosing.

The existence of vitamin D receptor (VDR) gene polymorphisms, which can affect the metabolism and/or transport of vitamin D, may also contribute to the response variation. A previous study investigated the effect of genetic variation in the vitamin D pathway on pulmonary tuberculosis patient’s response to four bi-weekly doses of 3500 µg vitamin D supplementation^(39)^. They identified novel variants in the VDR and CYP27B1 genes which modified the effect of the intervention on their primary outcome: accelerated sputum culture conversion^(39)^. Furthermore, single-nucleotide variations in CYP2R1 and CYP24A1 have been associated with lower levels of serum 25(OH)D and asthma severity^(40)^. A potential alternative explanation for the lower-than-expected increase in plasma 25(OH)D concentration is that compliance may not have been reported accurately by participants.

The key finding of the present study was that vitamin D supplementation was effective in a small but statistically significant improvement in the lung function marker: ratio FEV_1_:FVC. The ratio of FEV_1_:FVC is commonly used as a lung function marker in asthmatics^(41,42)^ and in vitamin D research related to lung function in asthma^(43–46)^. The increase in lung function measured by FEV_1_:FVC ratio was significantly higher in the intervention group compared with the placebo group, which suggests that daily supplementation with 125 µg vitamin D exerts a slight additional effect on lung function. This result differs from a previous study that investigated the effect on lung function measured by FEV_1_:FVC in asthmatics^(45)^. No significant effect of vitamin D supplementation was observed on lung function (*P* = 0·07). However, the dosing strategy used by this study included a bolus 5000 µg dose of vitamin D followed by monthly 2500 µg doses. This is a daily dose equivalent of approximately 82·5 μg of vitamin D, which is lower than the present study. Whilst the vitamin D treatment group saw a small but statistically significant increase in the ratio of FEV_1_:FVC, there was a decrease in FEV_1_ and FVC. A 10 % change in FEV_1_ from baseline is considered clinically significant^(47)^. This change was not realised in the vitamin D treatment group in the present study which may impact the clinical significance of the findings despite the statistically significant change in the ratio of FEV_1_:FVC. However, changes in both FEV_1_ and FVC can be affected by individual demographics such as age and sex^(48)^; therefore, there is a need for future studies to account for these factors. It would also be beneficial to have consistency across research studies in the exact lung function parameters reported. The present study utilised a per-protocol analysis, with five participants excluded from the analysis due to baseline data only, being available for these participants. As the present trial was analysed by comparison of baseline to post-intervention differences, this does not lend itself well to intention-to-treat analysis for a mixed model. However, intention-to-treat analysis using a ‘last observation’ single imputation showed that there was no significant change in FEV1, FVC or ratio FEV_1_:FVC from baseline to post-intervention between the vitamin D intervention and placebo group.

Additionally, in the present study, it was observed that baseline plasma 25(OH)D concentration influenced participant’s response to the intervention. There was a strong, inverse association between baseline 25(OH)D concentration and the mean change (week 0–week 12) in plasma 25(OH)D concentration. The improvement in vitamin D status in the vitamin D group was associated with a strong, significant inverse correlation between mean change in plasma 25(OH)D concentration and mean change in plasma IL-10 concentration from week 0 to week 12. IL-10 has been described as an anti-inflammatory cytokine and acts as a feedback regulator for the immune response by inhibiting inflammatory cytokine production^(49)^. Baseline levels of IL-10 can be used to predict improvement in lung function in patients supplemented with a single 7500 µg vitamin D dose^(44)^. This highlights IL-10 as an important candidate in future mechanistic studies investigating the role of vitamin D in asthma. Although there was no significant increase in plasma IL-10 levels in the present study, this cytokine should be explored in future studies as the variability in response to supplementation seen in the present study may have affected results.

It was demonstrated in this study that although 125 μg of vitamin D supplementation daily for 12 weeks significantly raised vitamin D levels, it was not adequate to increase overall vitamin D levels beyond the sufficiency threshold in the asthmatic population. The 2016 SACN report on vitamin D proposed a reference nutrient intake of vitamin D at 10 µg/d for the general population aged over 4 years. This is more than ten times lower than the supplement regimen used in the present study; therefore, it may be necessary for the current recommendation to be reconsidered for the winter months in the asthmatic population. Despite the high daily dose supplemented in the present study, none of the participants’ plasma 25(OH)D concentrations reached close to toxic levels. There is a possibility of reverse causation meaning that low plasma 25(OH)D concentrations are a consequence of the asthma disease state itself with chronic inflammation being associated with decreased serum 25(OH)D concentrations^(50)^. However, it is not believed to be the case in the present study where there was no effect of the supplement regimen raising plasma 25(OH)D concentrations on inflammatory biomarkers.

The findings from the present study are highly relevant to healthcare in the UK as widespread utilisation of vitamin D supplements may lead to improvement in vitamin D status. Furthermore, the population of South Asian adults in the UK are at further risk of deficiency with the majority failing to reach sufficient levels^(51)^. This is of particular concern during the winter months with median (IQR) 25(OH)D concentrations being 14·5 nmol/l (10·0–20·3 nmol/l) for South Asian adults compared with 47·3 nmol/l (29·0–59·3 nmol/l) in Caucasian adults. This highlights the necessity for further assessment of risk in asthmatic adults who may have more than one risk factor for vitamin D deficiency.

Caution is necessary for recommending daily vitamin D supplementation above the current reference nutrient intake due to safety concerns. However, the European Food Safety Authority determined a no-observed-adverse-effect-level of 250 µg/d^(52)^ with SACN setting an upper limit for vitamin D of 100 µg/d^(13)^. The safety of this limit has been questioned with a recent meta-analysis and systematic review of twenty-two clinical trials including over 12, 000 participants supplementing 80–100 μg/d vitamin D in trials lasting over 6 months^(53)^. The systematic review and meta-analysis found supplemental doses of vitamin D at this level may increase the risk of hypercalcaemia, falls and hospitalisation in some individuals^(53)^. This highlights the necessity of using caution when designing trials supplementing vitamin D at daily doses higher than the recommended 10 μg/d for over 6 months. The present study had no adverse events, although only lasted 12 weeks. In a clinical setting, it may be necessary to exceed the upper limit for vitamin D as the potential benefits of the supplementation may outweigh the possibility of adverse effects. Strict and rigorous reporting of safety-related outcomes must be considered throughout clinical trials.

There were limitations in the present study including only sufficiently being powered for the primary outcome of lung function parameters. Caucasians represented 96 % of the participant sample in the present study. As mentioned previously, there is evidence to suggest that asthmatics from Black and ethnic minorities may be at further increased risk of vitamin D deficiency and differences in vitamin D metabolism. Future research should consider these populations and VDR polymorphisms to investigate the benefits that vitamin D supplementation may provide. Single-nucleotide variations in genes involved in vitamin D metabolism have been associated with lower levels of serum 25(OH)D and asthma severity^(40)^. Furthermore, although the increase in FEV_1_:FVC was statistically significant, the increase was small which may impact the clinical significance of the findings; however, the study duration was 12 weeks and over a longer intervention period this increase may be greater; thus, future longer-term interventions are required.

In conclusion, we demonstrated that a 125 µg daily vitamin D supplement for 12 weeks was able to significantly increase the change in the ratio of FEV_1_:FVC compared with a placebo. This study supports previous research which suggests that a revision of current guidelines may be necessary for groups at higher risk of vitamin D deficiency, such as mild to moderate asthmatics. We have provided new insights on how daily dietary supplementation with vitamin D could lead to improved clinical outcomes, including lung function, and overall vitamin D status in mild to moderate asthmatic adults. Daily vitamin D supplementation at a dose greater than the current recommendation could be considered as an adjunct to current asthma therapy. This would be particularly important during the winter months in the UK when vitamin D deficiency and respiratory infections are prevalent. The findings also support the re-examination of recommendations for vitamin D supplementation, considering specific at-risk groups, to address the current prevalence of vitamin D deficiency in the asthmatic population.
