# A 12-week double-blind randomised controlled trial investigating the effect of dietary supplementation with 125 *µ*g/d vitamin D in adults with asthma – ERRATUM

**DOI:** 10.1017/S0007114524002472

**Published:** 2024-09-28

**Authors:** Stephanie Watkins, Tanja Harrison, Sohail Mushtaq

**Affiliations:** Faculty of Health, Medicine and Society, University of Chester, Chester CH1 4BJ, UK

In the original article the unit of measurement for Vitamin D was amended by the typesetter from IU to μg during the proofs stage, but the numbers were not updated to correspond to the change in unit.

The numbers have now been updated to show the correct µg of Vitamin D.

The Publisher apologises for this error.
